# Mendelian randomization reveals no associations of genetically-predicted obstructive sleep apnea with the risk of type 2 diabetes, nonalcoholic fatty liver disease, and coronary heart disease

**DOI:** 10.3389/fpsyt.2023.1068756

**Published:** 2023-02-09

**Authors:** Xiaoxu Ding, Lanqing Zhao, Xiangguo Cui, Li Qi, Yu Chen

**Affiliations:** ^1^Department of Otorhinolaryngology, Shengjing Hospital Affiliated With China Medical University, Shenyang, Liaoning, China; ^2^Department of Otorhinolaryngology, The First Hospital of China Medical University, Shenyang, Liaoning, China

**Keywords:** obstructive sleep apnea, type 2 diabetes, cardiometabolic diseases, Mendelian randomization, causal inference

## Abstract

**Background:**

Obstructive sleep apnea (OSA) has been reported to affect cardiometabolic diseases. However, whether such association is causal is still unknown. Here, we attempt to explore the effect of OSA on type 2 diabetes (T2D), nonalcoholic fatty liver disease (NAFLD) and coronary heart disease (CHD).

**Methods:**

Genetic variants associated with OSA were requested from a published genome-wide association study (GWAS) and those qualified ones were selected as instrumental variables (IV). Then, the IV-outcome associations were acquired from T2D, NAFLD and CHD GWAS consortia separately. The Mendelian randomization (MR) was designed to estimate the associations of genetically-predicted OSA on T2D, NAFLD and CHD respectively, using the inverse-variance weighted (IVW) method. We applied the Bonferroni method to adjust the p-value. Besides, MR-Egger regression and weighted median methods were adopted as a supplement to IVW. The Cochran's Q value was used to evaluate heterogeneity and the MR-Egger intercept was utilized to assess horizontal pleiotropy, together with MR-PRESSO. The leave-one-out sensitivity analysis was carried out as well.

**Results:**

No MR estimate reached the Bonferroni threshold (*p* < 0.017). Although the odds ratio of T2D was 3.58 (95% confidence interval (CI) [1.06, 12.11], IVW-*p*-value = 0.040) using 4 SNPs, such causal association turned insignificant after the removal of SNP rs9937053 located in FTO [OR = 1.30 [0.68, 2.50], IVW *p* = 0.432]. Besides, we did not find that the predisposition to OSA was associated with CHD [OR = 1.16 [0.70, 1.91], IVW *p* = 0.560] using 4 SNPs.

**Conclusion:**

This MR study reveals that genetic liability to OSA might not be associated with the risk of T2D after the removal of obesity-related instruments. Besides, no causal association was observed between NAFLD and CHD. Further studies should be carried out to verify our findings.

## Introduction

As a kind of severe sleep disorder, obstructive sleep apnea (OSA) is usually denoted as nocturnally repetitive episodes of breathing stops caused by upper airway collapse, resulting in mild to severe sleep deprivation and dysregulation of breathing, sleep, and blood pressure. It was estimated that at least 9% of the population suffered from it and its prevalence is increasing since 35% of individuals over 60 years of age suffer from it (1). Numerous patients remain underdiagnosed despite several available diagnostic tools and treatments (2). Continuous OSA status is usually accompanied by serious comorbidities through systemic inflammation and intermittent hypoxia (3). Besides, OSA is affected by multiple risk factors such as obesity, male sex, family history of OSA, high age and problems of upper airway flow or jaw anatomy (4).

Consequently, OSA burdens the public with increased mortality (5), which is caused by many cardiometabolic comorbidities including an increased risk of coronary heart disease (CHD), nonalcoholic fatty liver disease (NAFLD) (6), type 2 diabetes (T2D) and its complications (7, 8). Therein, the interplay between T2D and NAFLD has been reported where the genetically-predicted NAFLD could increase the risk of T2D and vice versa (9). Also, NAFLD and T2D could increase the risk of CHD as well (10, 11). However, whether the observed association is causal is still unknown and can be biased by potential confounders like socioeconomic status.

As a popular method of causal inference in molecular epidemiology, Mendelian randomization (MR) uses genetic variants as instrumental variables to detect the existence of causation and estimate its magnitude (12). It can simulate a randomized trial as the allocation of genetic variants at conception is random. Nowadays, it has made great contributions to causal inference, such as ruling out the association of genetically-predicted serum HDL with the risk of myocardial infarction (13). Thanks to the accumulating genome-wide association studies (GWAS), the summary statistics of the association between genetic variants and phenotype can be accessed much easier. However, it is still unknown whether OSA can lead to deleterious consequences in a causal setting. A recent MR study indicated that there was no causal association between sleep duration and glycemic traits (14), and our recent publication suggested that genetic susceptibility to OSA cannot affect the risk of Alzheimer's disease and Parkinson's disease (15). Additionally, a recent OSA GWAS only explored its causal relationship with body mass index (BMI) (16). Besides, there were no other MR studies focused on OSA.

We attempted to evaluate the associations of genetically-predicted OSA with T2D, CHD and NAFLD where OSA is the exposure and the remaining diseases are outcomes, hoping to disentangle their complex causal relationship.

## Methods

### Data description and study design

We retrieved data from the OSA GWAS using the FinnGen study (16). The GWAS summary statistics were extracted from it where 16,761 OSA patients and 201,194 controls were included in this FinnGen study. The nationwide health registries were used to identify OSA cases where the diagnosis of OSA was based on ICD-codes (ICD-10: G47.3, ICD-9: 3472A). The ICD-10 data were collected from the Finnish National Hospital Discharge Registry and the Causes of Death Registry. Several indicators were involved in diagnosing OSA, namely subjective symptoms, clinical examination and sleep registration applying AHI ≥ 5/h or respiratory event index (REI) ≥ 5/h. The GWAS analysis was performed using SAIGE, applying saddle point approximation (SPA) to calibrate unbalanced case-control ratios (17). Analyses were adjusted for age, sex, genotyping chip, genetic relationship and first 10 principal components.

We extracted IV for CHD from the Coronary ARtery DIsease Genome wide Replication and Meta-analysis (CARDIoGRAM) plus the Coronary Artery Disease (C4D) Genetics Consortium, with 60,801 cases and 123,504 controls adjusting for sex and age (18). The GWAS summary statistics of T2D were obtained from a European study with 62,892 cases and 596,424 controls, with adjustment of sex, age and study-specific information (19). The NAFLD GWAS consisted of 1,483 European NAFLD cases and 17,781 matched controls, adjusting for the first five principal components in multiple logistic regression (20).

Here, we appraise the effect of genetically-predicted OSA on three predefined outcomes, which consisted of three main previously-reported cardiometabolic comorbidities, including coronary heart disease, type 2 diabetes (21) and nonalcoholic fatty liver disease. There was no sample overlapping between exposure and outcome as the outcomes' GWAS data contained no FinnGen samples (22). For OSA cases, the median age was 58.9 years, the median BMI was 31.72 kg/m^2^, and the proportion of the males was 63.0%. For NAFLD cases, the median age was 50.1 years, the median BMI was 35.19 kg/m^2^ and the proportion of males was 52.7%. The samples of OSA, NAFLD and T2D were all of the European ancestry while the majority of CHD were of European ancestry (77%). Although we cannot give precise estimates on baseline characteristics of CHD and T2D, the two studies have adjusted for age, sex, and population stratification in the analyses. Thus, the bias caused by the imbalance of these distribution characteristics should not have a main impact on the MR results.

### Instrumental variable selection

**Figure 1:**
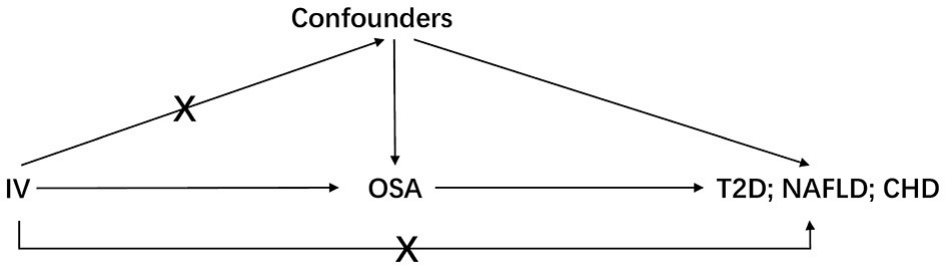
The basic assumptions of Mendelian randomization. IV is instrumental variable; OSA is obstructive sleep apnea; T2D is type 2 diabetes; NAFLD is nonalcoholic fatty liver disease; CHD is coronary heart disease.

Three basic assumptions should be satisfied in MR analysis: (1) The genetic variant should have a strong link to exposure; (2) There are no other potential confounders associated with the genetic variant; (3) The association of the genetic variant with the outcome can only be mediated *via* the way of exposure (Figure 1). Five genetic variants were reported to be associated with OSA by Strausz et al. (16). Considering its low minor allele frequency (MAF = 0.005), the single nucleotide polymorphism (SNP) rs185932673 was removed in the subsequent analyses. Generally, hypothesis 2 for MR analysis is untestable since we cannot determine all the potential confounders. Thus, we searched all 4 SNPs' associations in the open GWAS database (https://gwas.mrcieu.ac.uk/) to identify potential confounded associations and found that the SNP rs9937053 was strongly associated with BMI. Besides, the SNP rs9937053 was an intron in FTO, a well-established gene associated with obesity (23), and this SNP was marginally associated with OSA after adjustment of BMI (adjusted *p* = 0.04). Thus, we considered performing MR analyses with or without it in our study to assess the impact of the obesity-associated gene on the results. Therein, we were mainly focused on the results of MR analysis without SNP rs9937053. All the remaining SNPs displayed a genome-wide significance (*p* < 5 × 10^−8^) and a high imputation quality (INFO > 0.9). When the information of one SNP is missing the outcome, we would use another SNP in high linkage disequilibrium with it as the proxy using a predefined threshold LD r2 > 0.8. It should be noted that only SNP rs10507084 remained significant after adjusting for BMI and a single IV-based MR analysis was conducted using it as well.

### Statistical analysis

Before MR analysis, each IV's F-statistic was calculated using the formula as follows: F = $\frac{beta^{2}}{se^{2}}$. Here, beta represents the effect size of SNP on exposure and se is its corresponding standard error (24).

Besides, the general F-statistic was calculated as well: F = $\frac{N-k-1}{k}\times \frac{R^{2}}{1-R^{2}}$. The N is the sample size of exposure, and k is the number of used IVs. R^2^ represents the exposure's genetic variance explained by IVs.

The Wald ratio estimation was used to calculate the association of genetically-predicted OSA with the outcome for each SNP and the inverse-variance weighted (IVW) method was utilized to synthesize each SNP's casual estimation. Cochran's Q value was used to assess the heterogeneity and we would adopt a multiplicative random effect (MRE) model if the heterogeneity exists. Otherwise, we would combine the results using a fixed effect model. Besides, two other methods, including MR-Egger and weighted median, would also be adopted as a supplement to IVW.

Horizontal pleiotropy is a major issue in MR analysis and it should be sophisticatedly addressed. In our study, two methods were adopted, namely, MR-Egger intercept (25) and Mendelian randomization pleiotropy residual sum and outlier (MR-PRESSO) (26). For the MR-Egger intercept, there should be no difference between 0 and it if no horizontal pleiotropy exists. If the intercept significantly differs from 0, we assume there is horizontal pleiotropy and the results should be corrected by MR-Egger regression and be interpreted carefully. The MR-PRESSO is an effective method to detect outliers that might introduce horizontal pleiotropy into MR analyses using the residual sum. The MR analysis was performed using the R package “TwoSampleMR” (27) and “MRPRESSO” (26).

### Sensitivity analysis and power calculation

The sensitivity analysis was mainly carried out using a leave-one-out sensitivity analysis where each SNP was removed and the remaining SNPs were assumed as the IVs to estimate the associations of genetically-predicted exposure with the outcomes. If an SNP was detected as a driving IV obviously, we would drop it in the MR analysis. Such a “leave-one-out” method was used to judge whether the causal conclusion was robust to the outlier. This analysis was performed using the R package “TwoSampleMR” (27). We applied the mRnd to statistical power calculation (Power calculations for Mendelian Randomization) (https://cnsgenomics.shinyapps.io/mRnd/) (28).

## Results

Four genetic variants were used as eligible IVs in this MR analysis (Table 1). The general and each IV's F were all greater than the empirical threshold 10.

**Table 1:** Instrumental variables of obstructive sleep apnea.

**SNP**	**A1**	**A2**	**EAF**	**BETA**	**SE**	**P**	**F**
^*^rs9937053	G	A	0.43	0.104	0.012	4.30 × 10^−16^	82
#rs10507084	C	T	0.18	0.104	0.016	2.80 × 10^−11^	42
rs4837016	G	A	0.47	−0.073	0.011	1.50 × 10^−08^	44
rs10928560	C	T	0.18	−0.083	0.014	2.80 × 10^−08^	36

SNP is the rsID of genetic variants; A1 is the effect allele; A2 is the other allele; EAF is the effect allele frequency; BETA is the effect size of A1 on the exposure; SE is the standard error of beta; P is the p value of beta; F is the F statistics.

* rs9937053 is an obesity-related SNP and was removed in the main MR analysis. #rs10507084 is the only genome-wide significant SNP after adjusting for BMI and was used in the single IV analysis.

In the main analysis, three SNPs were used, including rs10507084, rs4837016 and rs10928560. This analysis suggested genetic susceptibility to OSA cannot affect the risk of T2D [OR = 1.30 [0.68, 2.50], IVW *p* = 0.432], NAFLD [OR = 0.65 [0.18, 2.37], IVW *p* = 0.513] and CHD [OR = 0.93 [0.45, 1.91], IVW *p* = 0.842] (Supplementary Figures 1–3). The leave-one-out sensitivity analyses suggested that the SNP rs10928560 might drive the main estimates among the 3 SNPs (Supplementary Figures 1B, 3B).

**Figure 2:**
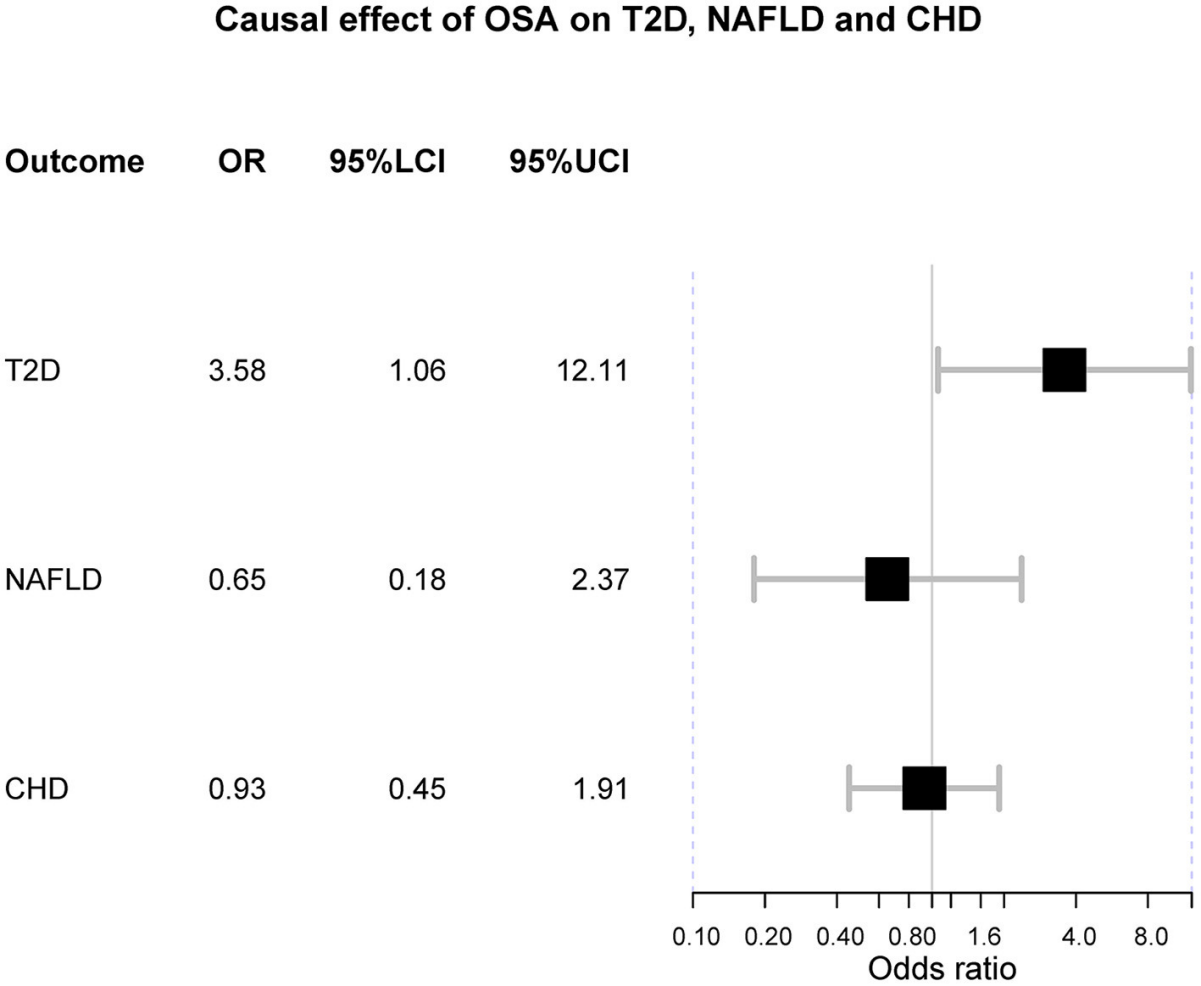
The forest plot of Mendelian randomization. OSA is obstructive sleep apnea; T2D is type 2 diabetes; NAFLD is nonalcoholic fatty liver disease; CHD is coronary heart disease; OR is odds ratio; 95%LCI is the lower limit of 95% confidence interval of OR; 95%UCI is the upper limit of 95% confidence interval of OR.

**Figure 3:**
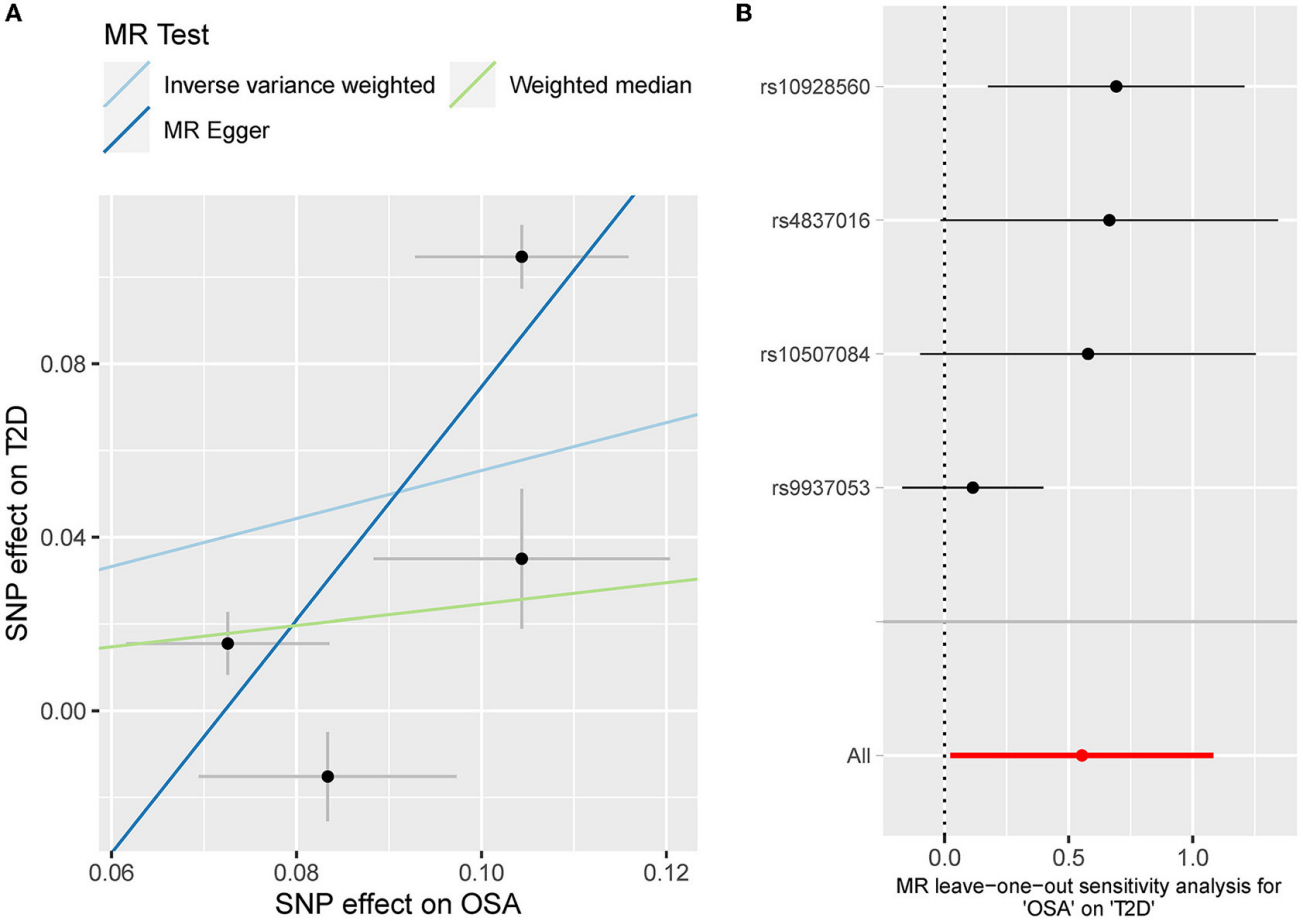
**(A)** The scatterplot of OSA-T2D results. Different colors represent different methods and each point is a single nucleotide polymorphism. The horizontal and vertical lines of each point represent the 95% confidence interval of the effect size. **(B)** The leave-one-out-sensitivity forest plot of OSA-T2D results.

The odds ratio of T2D was 3.58 [95% confidence interval (CI) [1.06, 12.11], IVW-*p* = 0.040] per 1-unit increase in log OR of OSA (Figures 2, 3A). From Figure 3A, the results of IVW and weighted median were similar while they were different from that of MR-Egger. However, there was significant heterogeneity (Cochrane's Q = 90.56, Q *p* = 1.66 × 10^−19^) and outliers were detected by MR-PRESSO. Besides, there was no pleiotropy by MR-Egger intercept (intercept = −0.19, se = 0.12, *p* = 0.252), thus, the results of IVW and weighted median should be more plausible. After the removal of outliers (SNP rs10928560 and rs9937053) in MR-PRESSO, the corrected OR was 2.16 [95%CI [1.08, 4.34], *p* = 0.030] per 1-unit increase in log OR of OSA. Also, the weighted median suggested a causal relationship between OSA and T2D [OR = 1.76 [1.15, 2.71], weighted median *p* = 0.010]. All the evidence indicated that the liability to OSA could elevate the risk of T2D.

**Figure 4:**
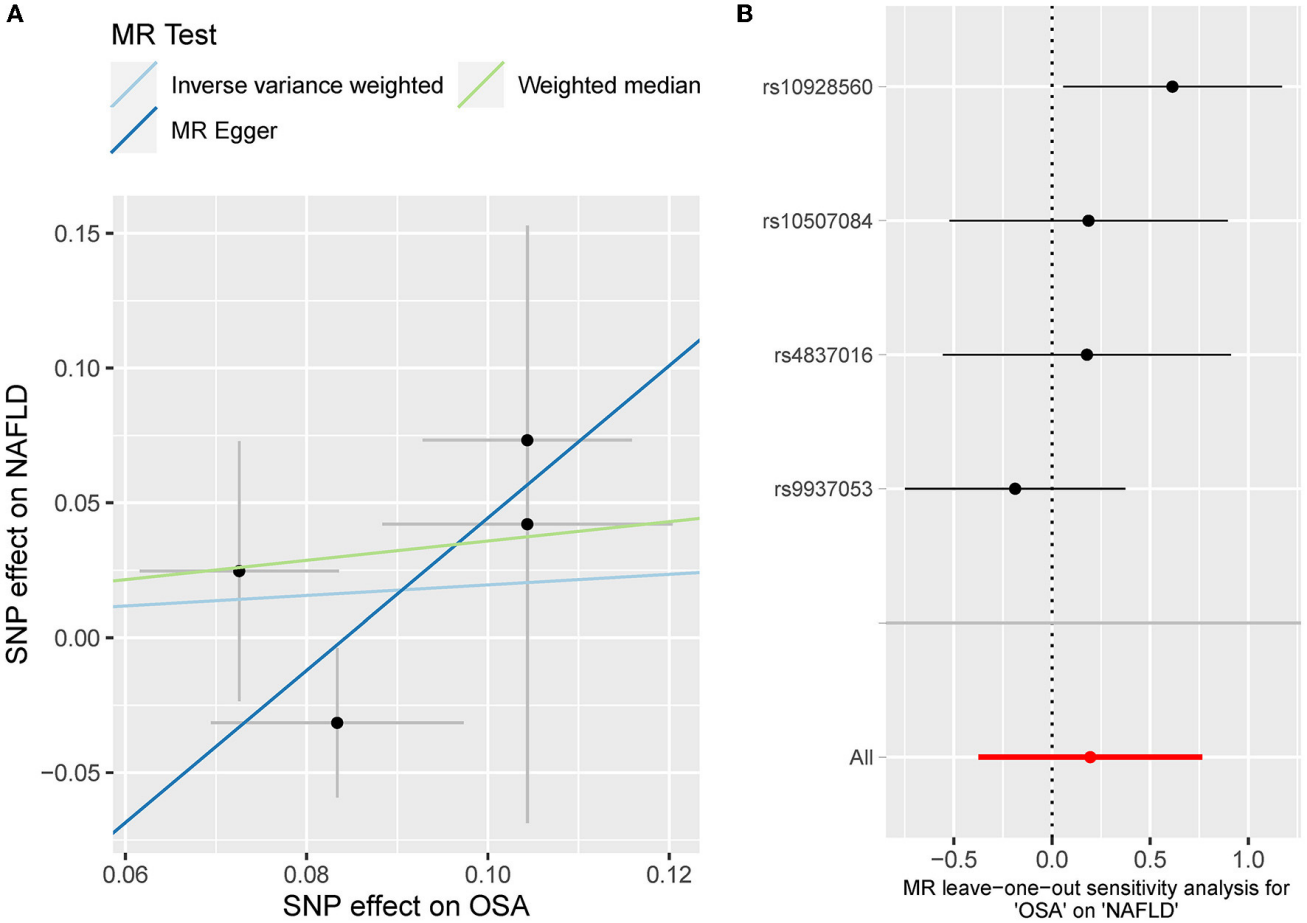
**(A)** The scatterplot of OSA-NAFLD results. Different colors represent different methods and each point is a single nucleotide polymorphism. The horizontal and vertical lines of each point represent the 95% confidence interval of the effect size. **(B)** The leave-one-out-sensitivity forest plot of OSA-NAFLD results.

**Figure 5:**
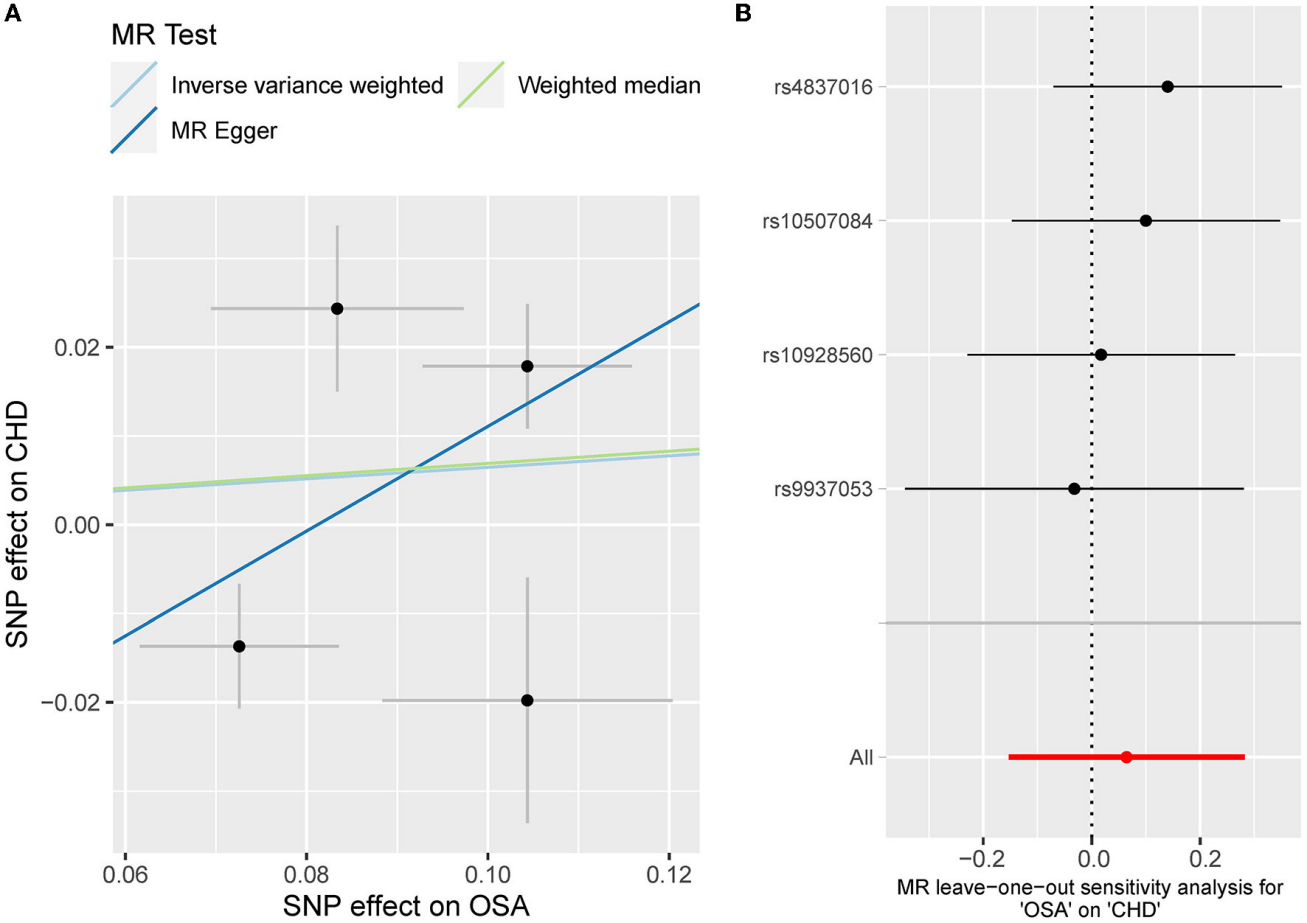
**(A)** The scatterplot of OSA-CHD results. Different colors represent different methods and each point is a single nucleotide polymorphism. The horizontal and vertical lines of each point represent the 95% confidence interval of the effect size. **(B)** The leave-one-out-sensitivity forest plot of OSA-CHD results.

Besides, the MR results indicated that genetically-predicted OSA could not be directly associated with the risk of NAFLD [OR = 1.57 [0.42, 5.82], IVW *p* = 0.501] (Figures 2, 4A) and CHD [OR = 1.16 [0.70, 1.91], IVW *p* = 0.560] (Figures 2, 5A). There was neither heterogeneity nor horizontal pleiotropy in OSA-NAFLD causal estimation (heterogeneity: Cochran's Q = 5.41, Q *p* = 0.144; pleiotropy: intercept = −0.24, se = 0.17, *p* = 0.302]. Also, no outliers were detected for it. While in OSA-CHD estimation, there was slight heterogeneity (Cochran's Q = 10.24, Q *p* = 0.017), and the corrected OR was 0.80 [95%CI [0.54, 1.20], *p* = 0.396] by MR-PRESSO. No horizontal pleiotropy was detected (intercept = −0.10, se = 0.04, *p* = 0.114).

After the removal of SNP rs9937053 located in FTO, the causal association with T2D turned insignificant [OR = 1.30 [0.68, 2.50], IVW *p* = 0.432]. No horizontal pleiotropy was observed (intercept = −0.01, se = 0.13, *p* = 0.960). However, there was heterogeneity (Cochrane's Q = 8.82, Q *p* = 0.012). And the weighted median also suggested a null association [OR = 1.42 [0.91, 2.21], weighted median *p* = 0.124]. Besides, no significant association was observed for CHD [OR = 0.93 [0.45, 1.91], IVW *p* = 0.842] and NAFLD [OR = 0.65 [0.18, 2.37], IVW *p* = 0.513]. No heterogeneity or horizontal pleiotropy was detected for OSA-CHD and OSA-NAFLD associations (Cochran's Q *p* > 0.05 and MR-Egger intercept *p* > 0.05). The single IV analysis revealed that genetic predisposition to OSA can increase the risk of T2D [OR = 1.36 [1.03, 1.80], *p* = 0.030] after adjustment of BMI while not for NAFLD [OR = 1.45 [0.21, 9.85], *p* = 0.704] and CHD [OR = 0.84 [0.66, 1.07], *p* = 0.153].

**Figure 6:**
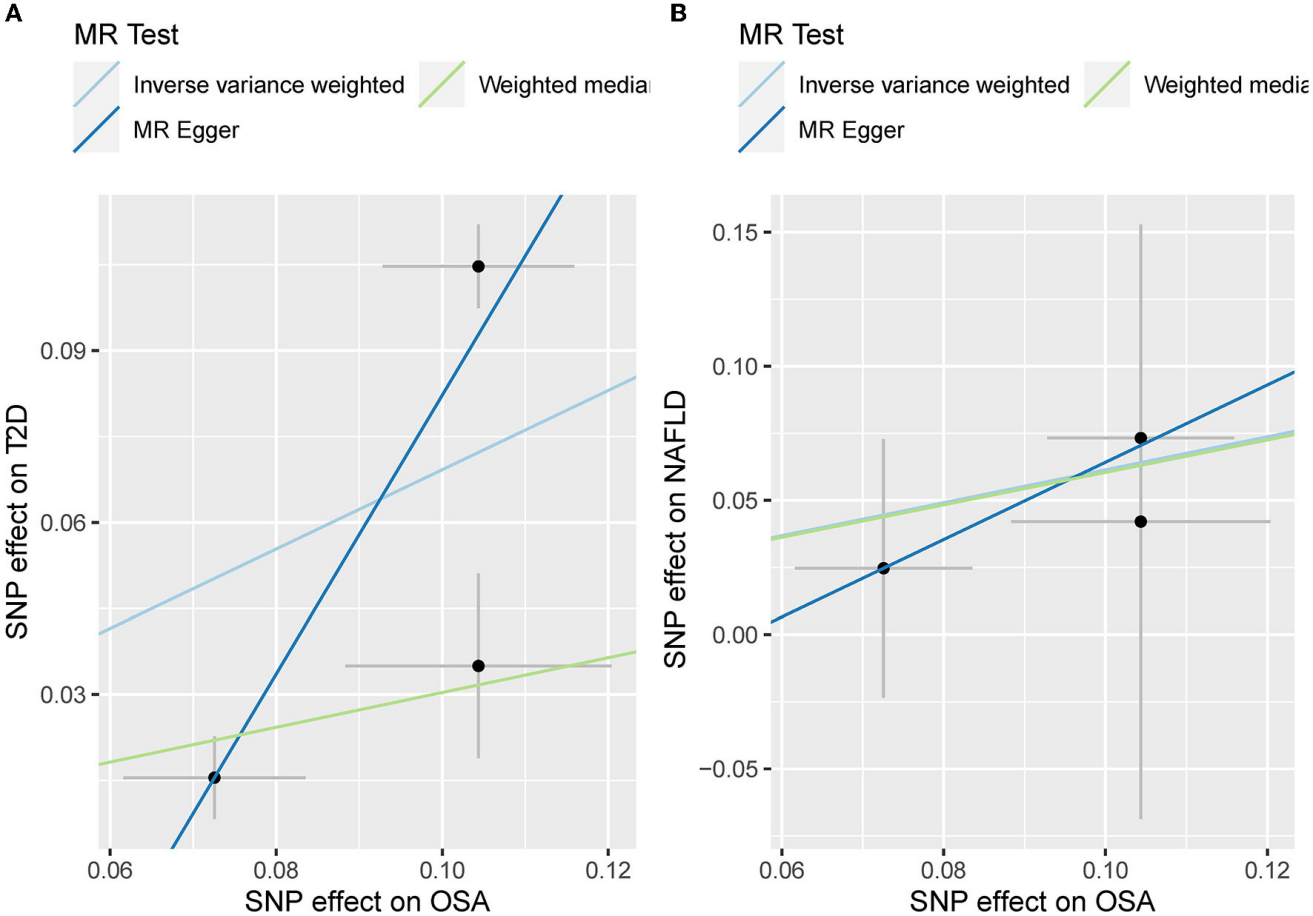
**(A)** The scatterplot of OSA-T2D results after removal of SNP rs10928560. Different colors represent different methods and each point is a single nucleotide polymorphism. The horizontal and vertical lines of each point represent the 95% confidence interval of the effect size. **(B)** The scatterplot of OSA-NAFLD results after removal of SNP rs10928560. Different colors represent different methods and each point is a single nucleotide polymorphism. The horizontal and vertical lines of each point represent the 95% confidence interval of the effect size.

**Figure 7:**
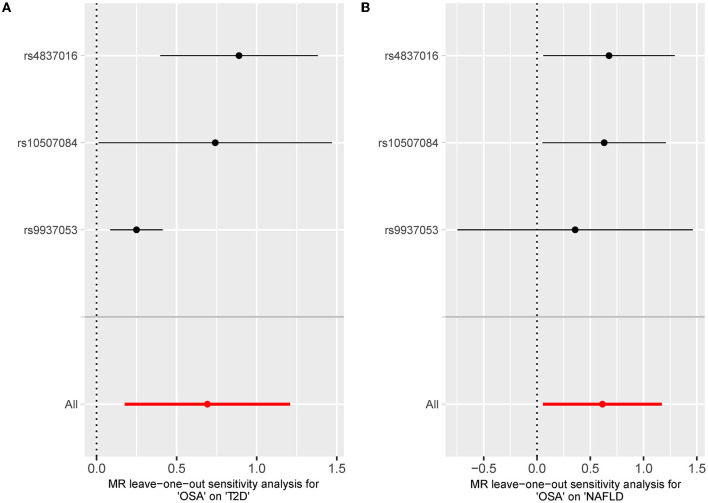
**(A)** The leave-one-out-sensitivity forest plot of OSA-T2D results after removal of SNP rs10928560. **(B)** The leave-one-out-sensitivity forest plot of OSA-NAFLD results after removal of SNP rs10928560.

The leave-one sensitivity suggested that the SNP rs10928560 might drive the causal estimation in OSA-T2D (Figure 3B) and OSA-NAFLD (Figure 4B) since its association was different from that of the other 3 SNPs. After removing rs10928560, the genetic predisposition to OSA would increase the risk of NAFLD [OR = 4.11 [1.14, 14.85], IVW *p* = 0.031] and T2D [OR = 4.92 [1.50, 16.18], IVW *p* = 0.009] (Figures 6, 7). And the estimates of IVW and weighted median methods are similar. We did not find SNPs that could drive the results in OSA-CHD estimation (Figure 5B). All statistical powers were above 80%.

Generally, we observed that genetic liability to OSA could increase the risk of T2D. However, such causation disappeared after the removal of SNP rs9937053 in FTO, an obesity-related gene. In leave-one-out sensitivity analysis, the genetic predisposition to OSA could elevate the risk of T2D and NAFLD after removing the driving SNP rs10928560. Besides, we did not discover any association between OSA and CHD. There was no other heterogeneity or horizontal pleiotropy detected in the analyses except the abovementioned.

## Discussion

Our MR study rules out the associations of genetically-predicted OSA with the risk of T2D, NAFLD, and CHD in a robust way and suggests that obesity-related genes might confound previous observational findings.

A recent dose-response meta-analysis indicated that a linear association should exist between OSA and T2D, and such an association was still significant after the adjustment of BMI (29). However, our MR analysis did not find a robust association, suggesting the previous finding might be mediated by obesity as the OSA-T2D association turned insignificant after the removal of SNP rs9937053 in FTO, an obesity-related gene. The relationship between OSA and BMI has been well recognized where a shared genetic background of them was reported and obesity played (30) an important role in the initiation of OSA (16). It should be noted that obesity is not always linked to an increased risk of cardiometabolic diseases and that accounting for metabolically healthy obesity (31) may provide clearer results about the proposed pathophysiological relationships. This might account for the heterogeneity in the MR results and why the OSA-T2D association turned insignificant after the removal of SNP rs9937053 in FTO. However, we cannot separate the effects of metabolically healthy and unhealthy obesity due to the unavailability of individual-level data, which needs further exploration. A higher risk for OSA could lead to an increased level of glycated hemoglobin (HbA1c) (32), and many studies reported that sleep restriction, intermittent hypoxia, and sleep fragmentation could lead to compromised insulin sensitivity in healthy individuals (33–35). Moreover, experimental studies have shown that intermittent hypoxia and sleep fragmentation can cause β-cell dysfunction or β-cell death (36). Additionally, it was reported that OSA can increase the T2D risk via the alteration of circadian rhythm (37). The possible intermediary pathways include increased sympathetic activity, altered hypothalamic-pituitary-adrenal axis, increased oxidative stress, activation of inflammatory pathways, and altered levels of circulating adipokines (38).

Many studies have implicated that the association of OSA with the initiation and development of NAFLD is independent of obesity or other shared risk factors (6). A recent large meta-analysis confirmed the strong association between the severity of OSA and steatosis (39). And previous large meta-analysis demonstrated that OSA was associated with higher triglycerides, low-density lipoprotein (LDL), and total cholesterol concentrations, as well as lower HDL concentrations after analyzing 107 datasets with over 18,000 patients (40). All the evidence corroborated the strong association between OSA and NAFLD. Our MR analysis found a high risk of OSA could elevate the risk of NAFLD after removing the SNP rs10928560 which could drive the result. This result further lent support to the OSA-NAFLD association. The OSA usually leads to sleep fragmentation, intrathoracic pressure swings, hypercapnia and intermittent hypoxia (41, 42). Rodent models suggested that the hypoxic status associated with OSA could play an important role in the development of dyslipidemia in OSA. Chronic intermittent hypoxia in obese mice could increase liver triglyceride concentration (43), promote hepatic lipid biosynthesis (43), and in lean mice, it could elevate total cholesterol and LDL concentrations (44), and induce atherosclerosis (45). These results help to explain why the genetic predisposition to OSA can increase the risk of NAFLD. However, NAFLD is also not homogenous regarding its pathophysiology and particularly genetically-induced fatty liver is, in most cases, not associated with an increased cardiometabolic risk (46). The innate heterogeneity of NAFLD might lead to opposite effects caused by OSA, and these effects might cancel out each other leading to a null association.

We did not discover a causal relationship between OSA and CHD, and this result is inconsistent with many previous studies where that suggested a high risk of OSA could elevate the risk of CHD (47). Also, a recent meta-analysis indicated OSA, especially severe OSA, is associated with reduced coronary flow reserve (48). Recently, Li et al. performed an MR analysis and found that genetically-predicted OSA should increase the risk of heart failure, however, the OSA-CHD association was not significant either. Li et al. included the SNPs rs9937053 and rs185932673 in their MR analyses, however, we removed them to avoid the bias caused by horizontal pleiotropy and imprecise statistical estimates since the SNP rs9937053 is located in the FTO gene, which is closely associated with obesity and the allele frequency of SNP rs185932673 is extremely low (< 0.01), which might cause statistical issues. Thus, we removed them in the main analysis. Additionally, we also included the SNPs rs9937053 in the supplementary analyses and found that genetic susceptibility to OSA should increase the risk of T2D [OR = 3.58 [1.06, 12.11], IVW-*p* = 0.040], suggesting the FTO variant should have a great impact on the results. To give conservative results, we chose to remove two ineligible SNPs in the main analysis and obtained negative results. OSA is common in patients with heart failure, stroke, and atrial fibrillation (49). Many studies have emphasized nuclear factor (NF)-κB–mediated pathways where rapid reoxygenation at the end of apnea produces free radicals, accelerating the reaction of oxidative stress and up-regulation of nuclear factor-κB (50). The pathogenesis of OSA can be attributed to another molecular signature called increased catecholamines, which is consistent with perturbations in the autonomic nervous system. As explained in NAFLD, OSA could disturb lipid metabolism, elevate total cholesterol and LDL concentrations (44), and induce atherosclerosis (45). All these mean OSA should be associated with CHD no matter of epidemiological or experimental studies and we cannot rule out their causal relationship merely based on MR results. This MR analysis failed to detect such association, and several reasons might account for it: (1) The strict criteria of IV selection might reduce the statistical power and lead to an increased false negative rate. This is common in MR analysis; (2) We failed to observed the total effect of OSA on CHD, but we cannot disregard the possibility that OSA can lead to CHD *via* mediated ways, such as obesity, T2D, and NAFLD; (3) The direct and indirect effects can cancel out and further result in null association, possibly due to undetected mediators. Another important aspect is that the OSA might be caused by CHD or obesity, which might lead to the observed association between OSA and cardiometabolic diseases, however, the observed association did not indicate that OSA was a risk factor and such reverse causation for CHD-OSA cannot be assessed currently due to a lack of full summary statistics of OSA GWAS. These potential reasons warrant further investigations into the causal relationship between OSA and CHD.

Our MR study strengthens the OSA-T2D and OSA-NAFLD causal associations using a robust causal inference method. However, we have to clarify several limitations for future investigations: (1) Horizontal pleiotropy is the main issue in MR analysis and our study is no exception. We have applied MR-Egger interception and MR-PRESSO to evaluate it. Furthermore, we excluded the SNP rs9937053 in the FTO gene and reevaluate the result. It should be noted that the OSA-T2D association became insignificant after the removal of SNP rs9937053 and we deemed obesity might play an important role in OSA-T2D association. However, considering that SNP rs9937053 was still significantly associated with OSA after adjustment of BMI, we still included it in our main analysis. (2) Since OSA is a binary exposure, we cannot appropriately appraise the selection bias and exclusive restriction bias due to data limitations. (3) Neither multivariable nor mediation MR analysis can be performed to disentangle the mediation effects and we cannot include more genetic instruments because of the unavailability of the full summary GWAS statistics for OSA. (4) We are mainly focused on the European population and the generalizability of our conclusion is limited. We cannot easily expand our conclusion to other populations. (5) The relationship between the severity of OSA and other parameters cannot be assessed due to a lack of individual-level data. Overall, such negative results pinpointed that OSA might not directly affect T2D, NAFLD, and CHD if not using the SNP shared by OSA and obesity. Obesity might be a key factor that links OSA to T2D, NAFLD and CHD, which should be paid attention to in future clinical and scientific research work.

## Conclusion

This MR analysis indicated that genetically-predicted OSA might not affect the risk of type 2 diabetes, nonalcoholic fatty liver disease and coronary heart disease.

## Data availability statement

The original contributions presented in the study are included in the article/Supplementary material, further inquiries can be directed to the corresponding author.

## Author contributions

YC contributed to the study design, supervised the data analysis process, and mainly revised the manuscript. XD and LZ were responsible for data acquisition, statistical analysis, and data visualization. XD drafted the original manuscript. XC and LQ read and revised the original manuscript and gave substantial suggestions on statistics. YC takes responsibility for the integrity of the data and the accuracy of the data analysis. All authors have approved for the publication of this study.

## Supplementary material

Supplementary Figure 1 **(A)** The scatterplot of OSA-T2D results after removal of SNP rs9937053. Different colors represent different methods and each point is a single nucleotide polymorphism. The horizontal and vertical lines of each point represent the 95% confidence interval of the effect size. **(B)** The leave-one-out-sensitivity forest plot of OSA-T2D results after removal of SNP rs9937053.

Supplementary Figure 2 **(A)** The scatterplot of OSA-NAFLD results after removal of SNP rs9937053. Different colors represent different methods and each point is a single nucleotide polymorphism. The horizontal and vertical lines of each point represent the 95% confidence interval of the effect size. **(B)** The leave-one-out-sensitivity forest plot of OSA-NAFLD results after removal of SNP rs9937053.

Supplementary Figure 3 **(A)** The scatterplot of OSA-CHD results after removal of SNP rs9937053. Different colors represent different methods and each point is a single nucleotide polymorphism. The horizontal and vertical lines of each point represent the 95% confidence interval of the effect size. **(B)** The leave-one-out-sensitivity forest plot of OSA-CHD results after removal of SNP rs9937053.
