# Supplementary figures and images for: Mendelian randomization reveals no associations of genetically-predicted obstructive sleep apnea with the risk of type 2 diabetes, nonalcoholic fatty liver disease, and coronary heart disease
Source: Front Psychiatry. 2023 Feb 9;14:1068756. doi: 10.3389/fpsyt.2023.1068756 (PMC9949721; doi:10.3389/fpsyt.2023.1068756)

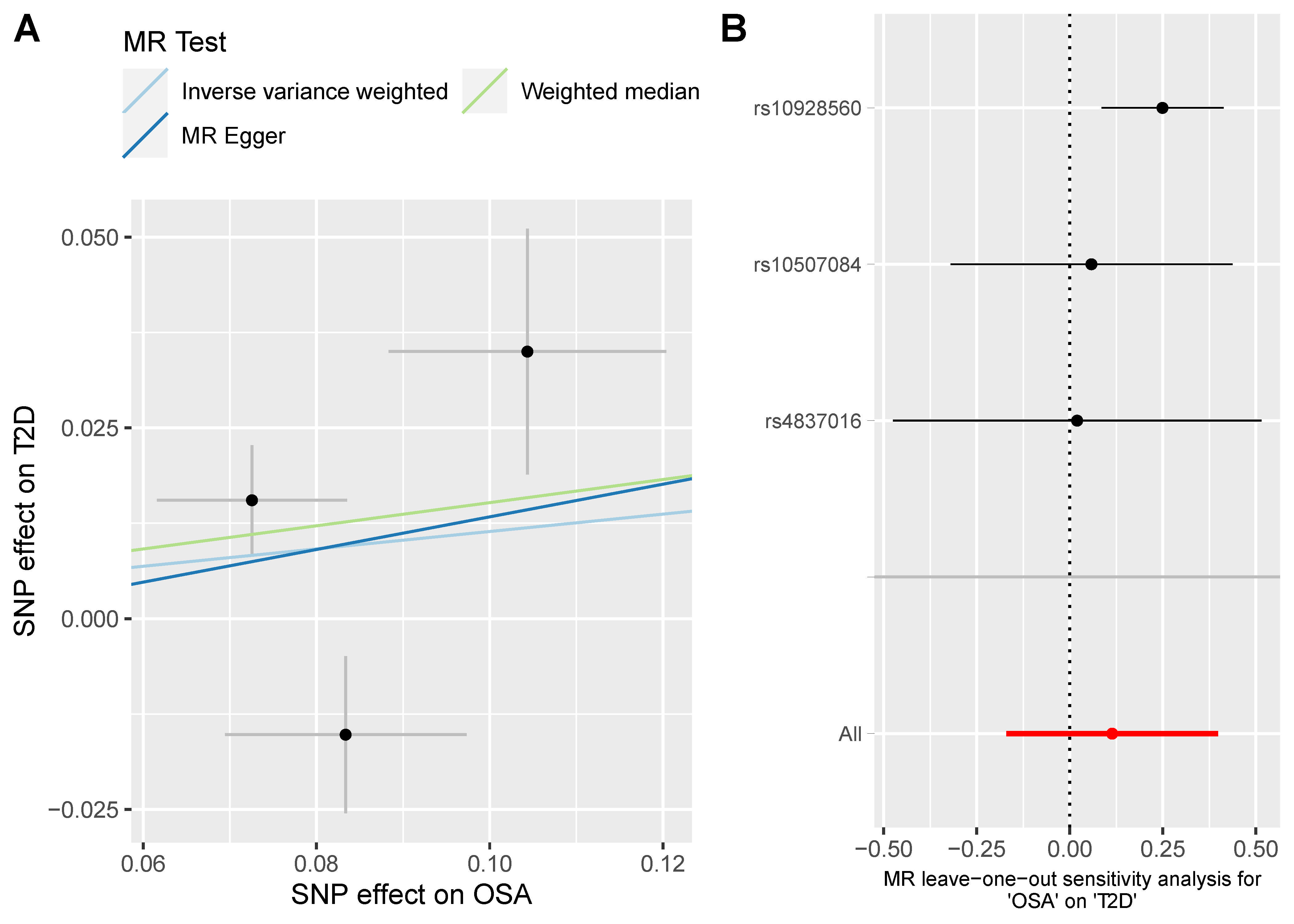

Supplement: Supplementary Figure 1 — (A) The scatterplot of OSA-T2D results after removal of SNP rs9937053. Different colors represent different methods and each point is a single nucleotide polymorphism. The horizontal and vertical lines of each point represent the 95% confidence interval of the effect size. (B) The leave-one-out-sensitivity forest plot of OSA-T2D results after removal of SNP rs9937053. [file Image_1.TIF]

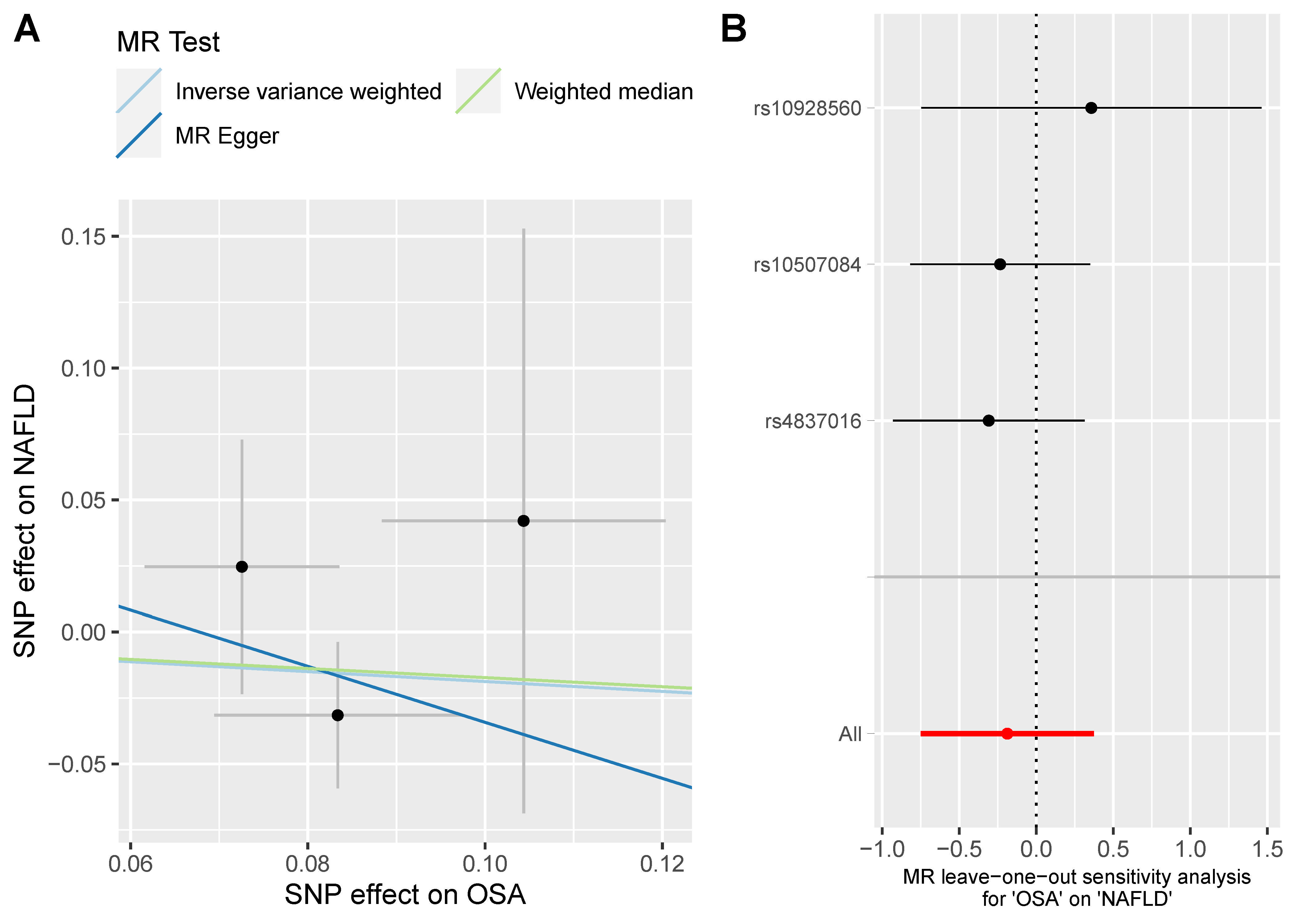

Supplement: Supplementary Figure 2 — (A) The scatterplot of OSA-NAFLD results after removal of SNP rs9937053. Different colors represent different methods and each point is a single nucleotide polymorphism. The horizontal and vertical lines of each point represent the 95% confidence interval of the effect size. (B) The leave-one-out-sensitivity forest plot of OSA-NAFLD results after removal of SNP rs9937053. [file Image_2.TIF]

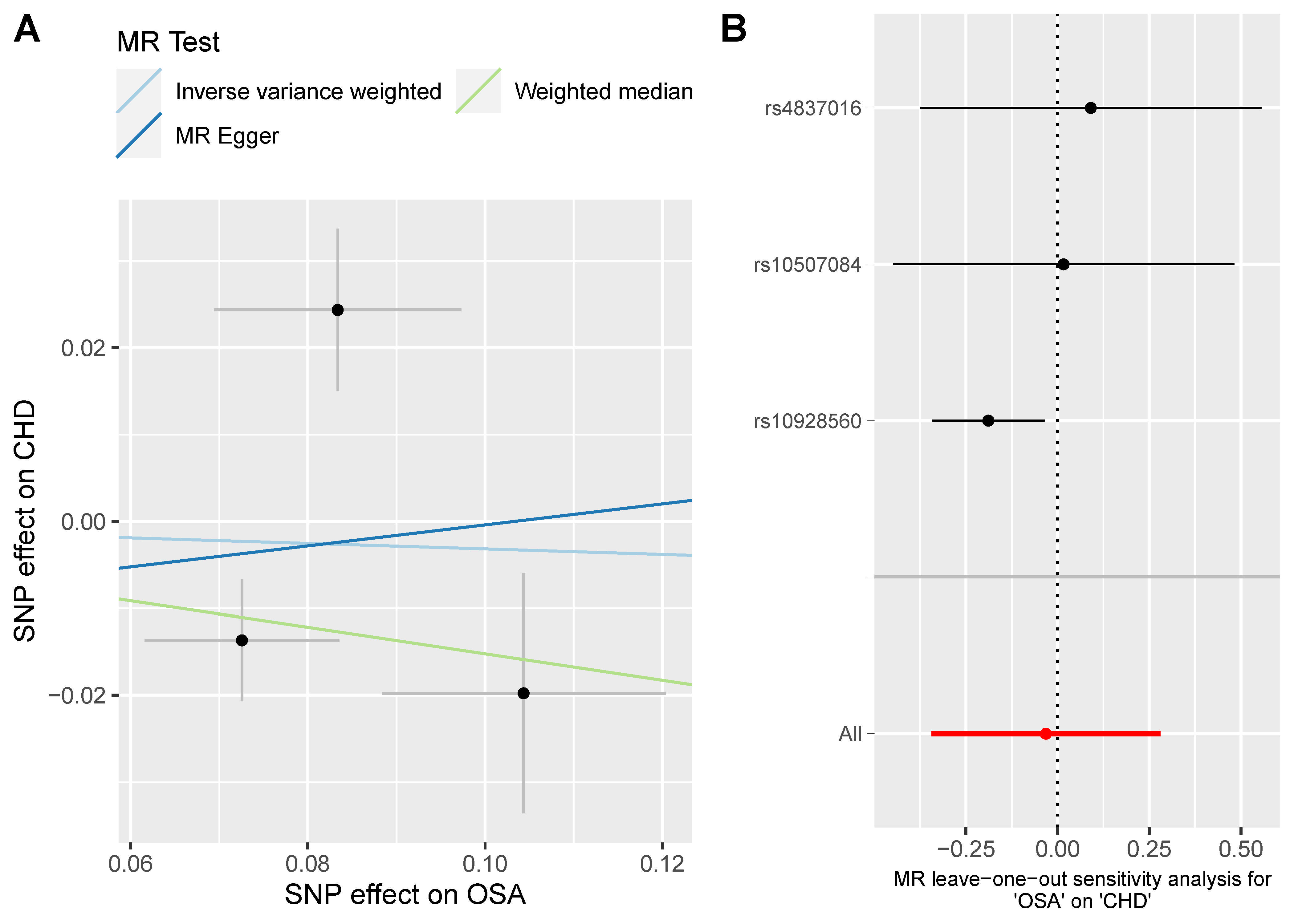

Supplement: Supplementary Figure 3 — (A) The scatterplot of OSA-CHD results after removal of SNP rs9937053. Different colors represent different methods and each point is a single nucleotide polymorphism. The horizontal and vertical lines of each point represent the 95% confidence interval of the effect size. (B) The leave-one-out-sensitivity forest plot of OSA-CHD results after removal of SNP rs9937053. [file Image_3.TIF]
